# The radiation adaptive response and priming dose influence: the quantification of the Raper–Yonezawa effect and its three-parameter model for postradiation DNA lesions and mutations

**DOI:** 10.1007/s00411-022-00963-9

**Published:** 2022-02-12

**Authors:** Krzysztof W. Fornalski, Łukasz Adamowski, Ludwik Dobrzyński, Rafał Jarmakiewicz, Aleksandra Powojska, Joanna Reszczyńska

**Affiliations:** 1 National Centre for Nuclear Research (NCBJ), ul. A. Sołtana 7, 05-400 Otwock-Świerk, Poland; 2 Faculty of Physics, Warsaw University of Technology, ul. Koszykowa 75, 00-662 Warsaw, Poland; 3 Department of Biophysics, Physiology and Pathophysiology, Faculty of Health Sciences, Medical University of Warsaw (WUM), ul. T. Chałubińskiego 5, 02-004 Warsaw, Poland

**Keywords:** Adaptive response, Radiation, Yonezawa effect, Priming dose, Challenging dose, Radioadaptation, Radiosensitivity, Radiation biophysics, Cancer physics, Lymphocytes

## Abstract

The priming dose effect, called also the Raper–Yonezawa effect or simply the Yonezawa effect, is a special case of the radiation adaptive response phenomenon (radioadaptation), which refers to: (a) faster repair of direct DNA lesions (damage), and (b) DNA mutation frequency reduction after irradiation, by applying a small priming (conditioning) dose prior to the high detrimental (challenging) one. This effect is observed in many (but not all) radiobiological experiments which present the reduction of lesion, mutation or even mortality frequency of the irradiated cells or species. Additionally, the multi-parameter model created by Dr. Yonezawa and collaborators tried to explain it theoretically based on experimental data on the mortality of mice with chronic internal irradiation. The presented paper proposes a new theoretical approach to understanding and explaining the priming dose effect: it starts from the radiation adaptive response theory and moves to the three-parameter model, separately for two previously mentioned situations: creation of fast (lesions) and delayed damage (mutations). The proposed biophysical model was applied to experimental data—lesions in human lymphocytes and chromosomal inversions in mice—and was shown to be able to predict the Yonezawa effect for future investigations. It was also found that the strongest radioadaptation is correlated with the weakest cellular radiosensitivity. Additional discussions were focussed on more general situations where many small priming doses are used.

## Introduction

The radiation adaptive response effect (called also a radioadaptation) is a biophysical phenomenon, which may occur in the organism irradiated to low doses of ionizing radiation. This effect connects the irradiation process with the adaptation to radiation, which causes faster DNA lesion repair and reduction of DNA mutation creation (Olivieri et al. 1984; Azzam et al. 1994; Wolff 1998; Feinendegen 1999; Tapio and Jacob 2007; Dimova et al. 2008; Mitchel 2009; Guéguen et al. 2019). However, because not all irradiation conditions induce radioadaptation (Mortazavi et al. 2003; Wójcik et al. 1996), and the effect shows strong individual variability (Bosi and Olivieri 1989), it can be very difficult to predict. Therefore, many scientific investigations in radiobiology and radiation biophysics aimed at understanding this phenomenon are still going on.

Till recently, it has been assumed that the radiation adaptive response effect occurred when enhanced repair mechanisms started after the creation of damage within the DNA chain (Mitchel 2009). Nowadays, new findings show that small radiation dose(s) can affect the expression of gene transcription (Sokolov and Neumann 2015). Exposure in proper conditions can produce “an alert, triggering and altering cellular responses to defend against subsequent high dose-induced damages, and accelerating the cell repair process. Moreover, the p53 signalling pathway was found to play critical roles in regulating DNA damage responses” (Hou et al. 2015) because “the p53 pathway is composed of a network of genes and their products that are targeted to respond to a variety of intrinsic and extrinsic stress signals that impact upon cellular homeostatic mechanisms that monitor DNA replication, chromosome segregation and cell division” (Harris and Levine 2005; Vogelstein et al. 2000). Therefore, the adaptive response mechanism may increase DNA repair up-regulation, leading to the reduction of postradiation mutation frequency (Dimova et al. 2008).

**Fig. 1:**
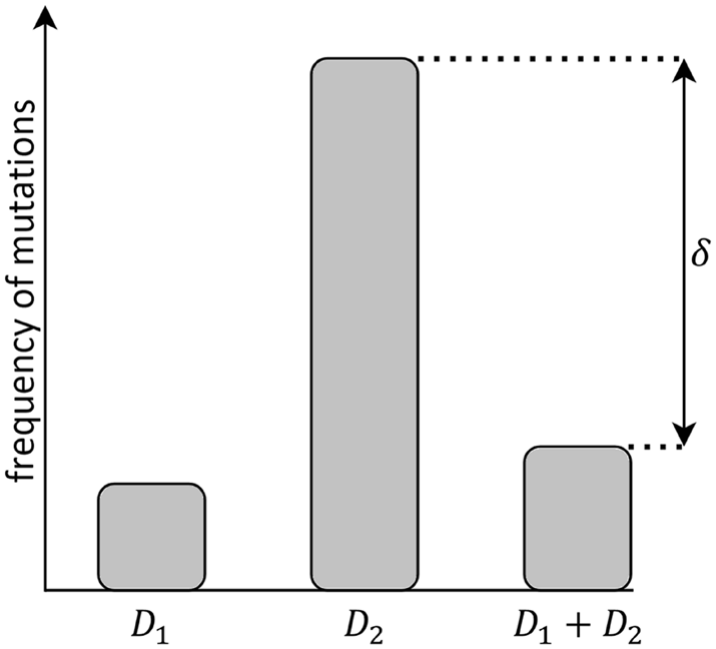
The scheme of the Yonezawa effect (also called the priming dose effect or the Raper-Yonezawa effect): the single small dose (*D*_1_) generates much less mutations than the single high dose (*D*_2_). However, when *D*_2_ follows *D*_1_ with some time distance between them (Δ*t*), the mutation (or lesion) frequency for that *D*_1_ + *D*_2_ total dose is lower than for single *D*_2_ by the exemplary value of *δ* = 0.73. This exemplary result was obtained using the input data: *D*_1_ = 1 UAD (Unit of Absorbed Dose), *D*_2_ = 5 UAD, Δ*t* = 3 UT (Unit of Time), *α*_0_ = 1 [UT^−3^ UAD^−2^], *α*_1_ = 1 [UAD^−1^], *α*_2_ = 0.7 [UT^−1^]. The parameter *δ* is therefore showing the percentage difference between the number of mutations (or lesions) generated by the single dose *D*_2_ (without the priming dose) and the combination of *D*_1_ + *D*_2_

There are two main processes of triggering the radiation adaptive response: via chronic irradiation or pulse single doses. In the first case, the effect can be observed e.g. within some people living in areas with a high background radiation (Scott et al. 2009; Dobrzyński et al. 2015a, 2015b). The second case is equivalent to the priming dose effect, also called the Yonezawa phenomenon (originally for mice) (Yonezawa et al. 1990, 1996; Matsumoto et al. 2004; Tapio and Jacob 2007), where the small priming dose (*D*_1_) received prior to the high challenging dose (*D*_2_) can reduce detrimental effects of the latter (Shadley and Wolff 1987; Wang et al. 2013; Toossi et al. 2016; Hauptmann et al. 2016). The exemplary scheme of this effect is presented in Fig. 1, where the difference between the *D*_1_ + *D*_2_ scheme (with time Δ*t* between doses) and the single *D*_2_ one is denoted as delta (*δ*) parameter, which is the main quantification of the generalized Yonezawa effect. One has to note, however, that the first observation of this effect (without deeper explanation) was conducted by Prof. Raper in 1940s during the Manhattan Project (Raper 1947; Cronkite et al. 1950).

The first theoretical explanation of this phenomenon was given by Dr Yonezawa and collaborators, who created the phenomenological multi-parameter model describing the effect (Smirnova and Yonezawa 2003, 2004). The approach proposed by Smirnova and Yonezawa is a deterministic model, describing the effect of ionizing radiation on the cells of four major hematopoietic lines: thrombocytes, lymphocytes, erythrocytes, and granulocythes. It divides cells into categories depending on the stages of development (bone marrow precursor cells, nondividing maturing cells, and mature blood cells) as well as on the level of damage (undamaged cells, damaged cells, and heavily damaged cells), and describes the relationships between those categories. To describe the damaging effect of ionizing radiation on the cells, the one-target-one-hit theory is used. The model consists of a system of differential equations with many unknown (free) parameters that must be determined based on experimental data. It allows the simulation of the effect of either chronic irradiation or pulse single doses. It should be noted that this model has limited application to certain types of cells (namely thrombocytes, lymphocytes, erythrocytes, and granulocythes only) and that the large number of equations and unknown (free) parameters make it hard to apply in practice.

The presented paper proposes a novel theoretical approach to the radiation adaptive response phenomenon, leading to the Yonezawa effect. This results in a three-parameter model, which works well after parametric quantification based on experimental radiobiological data.

The model is divided into two parts: in the first part the calculations focus on the reduction of DNA damage (lesions) in quite a short time after the irradiation; in the second part late effects, namely stable mutations, are considered. The latter case is much easier to consider in experimental radiobiological research.

## Adaptive response phenomenon

The proposed theoretical approach to the radiation adaptive response effect was originally presented a few years ago (Fornalski 2014) but its detailed biophysical explanation has been published very recently (Dobrzyński et al. 2019). Therefore, only a short and general mechanistic description will be presented here.

Generally, linear and non-linear effects can cause a response described by the specific hunchbacked shape (Feinendegen 2005, 2016) of time- and dose-related probability functions of the radioadaptation appearance, respectively:

1 $$ \left\{ {\begin{aligned} &{P\left( D \right) = \beta_{1} D^{\nu } e^{{ - \alpha_{1} D}}} \\ &{P\left( t \right) = \beta_{2} t^{\eta } e^{{ - \alpha_{2} t}} } \end{aligned} } \right., $$

where *D* and *t* denotes radiation dose and time after irradiation, respectively, and {*α*}, {*β*}, *ν*, *η* are free parameters. Both functions can be merged into a single, time- and dose-dependent, probability distribution of the adaptive response appearance after the single dose pulse *D* received *t* time ago (Fornalski 2014; Dobrzyński et al. 2019):

2 $$ p_{{{\text{AR}}}} = \alpha_{0} D^{2} t^{2} e^{{ - \alpha_{1} D - \alpha_{2} t}} , $$

where all parameters are positive ones while *ν* and *η* parameters were assumed to be equal to 2, which follows the result of pooled simplified quantification by Feinendegen (2016). Equation (2) represents the simplest version of the adaptive response per unit of time. Please note that the function *p*_AR_ reaches its maximum value for the dose *D*_max_ = 2*/α*_1_ and for the time *t*_max_ = 2*/α*_2_ after irradiation. In general, Eq. (2) can be rewritten in the continuous form as (Fornalski 2014):

3 $$ p_{{{\text{AR}}}} = \alpha_{0} \mathop \int \limits_{t = 0}^{\tau } \dot{D}^{2} \left( {\tau - t} \right)^{2} e^{{ - \alpha_{1} \dot{D} - \alpha_{2} \left( {\tau - t} \right)}} {\text{d}}t, $$

where *Ḋ* corresponds to the time-dependent dose-rate and *τ* is the cell’s (or the organism’s) age. In practice, however, Eq. (3) is usually used for chronic irradiation, where *Ḋ* is given by some dose distribution, *Ḋ*(*t*) (Dobrzyński et al. 2019). But in the biophysical calculations it is easier to apply Eq. (2) (or its combinations) (Dobrzyński et al. 2016; Fornalski et al. 2017; Fornalski 2019). When the dose distribution is discrete, namely single dose pulses *D*_*k*_ in time steps *k*, the dedicated form of Eq. (3)

4 $$ p_{{{\text{AR}}}} = \alpha_{0} \mathop \sum \limits_{k = 0}^{K} D_{k}^{2} \left( {K - k} \right)^{2} e^{{ - \alpha_{1} D_{k} - \alpha_{2} \left( {K - k} \right)}} , $$

can be used e.g. in Monte Carlo simulations (Fornalski 2014; Loan et al. 2019). Equation (4) shall be understood as: each dose *D*_*k*_ received *K–k* steps ago (*K* is the age) generates a single signal given by Eq. (2) extended over time, which is additive to the rest *K* − 1 signals described by an identical mathematical form (Fornalski 2014). The summation presented in Eq. (4) allows one to freely add every single adaptive response signal generated by each dose *D*_*k*_ under two conditions:

- the dose *D*_*k*_ is a short pulse with the duration of *t*_*Dk*_ ⟶ 0;
- the time interval between two consecutive doses is large enough, Δ*t* >> *t*_*Dk*_.

If some of the conditions presented above are not fulfilled, Eq. (3) shall be used instead. In particular, for two separate dose pulses, namely *D*_1_ and *D*_2_, which are received with the time interval Δ*t* > 0, one can simply denote the summarized probability distribution of the adaptive response as *p*_AR_(*D*_1_,*D*_2_,*t*) = *p*_AR_(*D*_1_,*t*_0_) + *p*_AR_(*D*_2_, *t*_0_ + Δ*t*). When both doses are received in the same time (Δ*t* = 0), one shall write *p*_AR_(*D*_1_,*D*_2_,*t*) = *p*_AR_(*D*_1_ + *D*_2_,*t*) because they can be simply treated as the single dose (*D*_1_ + *D*_2_).

## Kinetics of DNA lesions repair

Let us assume that the dose pulse, *D*, generates some number of fast and direct damage, *N*, called DNA lesions. Regarding the actual experimental findings (Rothkamm et al. 2007; Manning et al. 2013), this dependence is assumed to be linear with additional background metabolic lesions (for a zero-dose situation), therefore

5 $$ N = \mu_{0} + \mu_{1} D. $$

In other words, *N* from Eq. (5) represents the immediate number of physical damage (lesions) generated linearly by the dose *D*. All parameters {*μ*} are calibration parameters of Eq. (5), of which values are given in the literature e.g. in direct initial lesions testing (Ward 1995) and their background level. Especially, the parameter *μ*_0_ corresponds to the frequency of spontaneous lesions (without radiation), and *µ*_1_ is a linear slope. One has to note, however, that the free parameter *µ*_0_ is rather small (*µ*_0_ << *µ*_1_) and can be simply neglected. Exemplary values of {*μ*} parameters for damage in human lymphocytes after neutron irradiation equal *µ*_0_ = 0.0005 and *µ*_1_ = 0.832 (IAEA 2011; Słonecka et al. 2018).

The repair mechanisms start just after lesion (*N*) appearance, however, the probability of the adaptive response is strictly correlated with the number of repaired damage after a period of time, which can be presented as (Foray et al. 2005)

6 $$ {\text{d}}N = - N\, p_{{{\text{AR}}}} \,{\text{d}}t , $$

where *p*_AR_ is given by Eq. (2) for single dose pulse irradiation. It is assumed that this mechanism is the only method of repair after postradiation lesion appearance. Please note that Eq. (6) is the basis for the general function of the remaining number of lesions in an actual moment in time, *N*(*T*). The detailed calculations are presented in the Appendix 1.

**Fig. 2:**
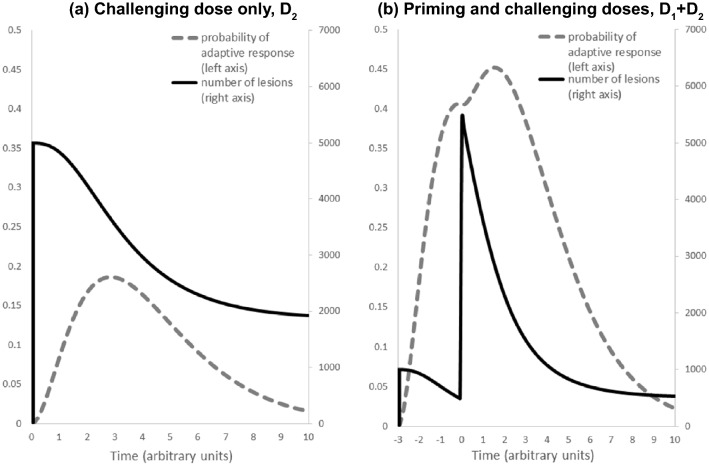
The time-dependent relations of the probability distribution of the adaptive response, *p*_AR_, and the number of postradiation lesions, *N*(*t*), given by Eq. (2) and Eqs. (21)–(22), respectively. Plot **a** presents a scenario where a single (reference) dose *D*_2_ is applied; one can observe the hunchbacked shape of the *p*_AR_ function and the decrease in the number of lesions, *N*(*t*). Plot **b** presents a scenario with a combination of doses *D*_1_ + *D*_2_, where the priming dose *D*_1_ is given prior to the challenging dose *D*_2_ (the latter received in the same moment as in the plot **a**)); one can observe that the priming dose increased the probability of successful repair (enhanced value of *p*_AR_) which causes a stronger decrease in the number of lesions, *N*(*t*). All input data were the same as in Fig. 1. The variable *t* is the global time where *t* = 0 corresponds to the moment of *D*_2_

**Fig. 3:**
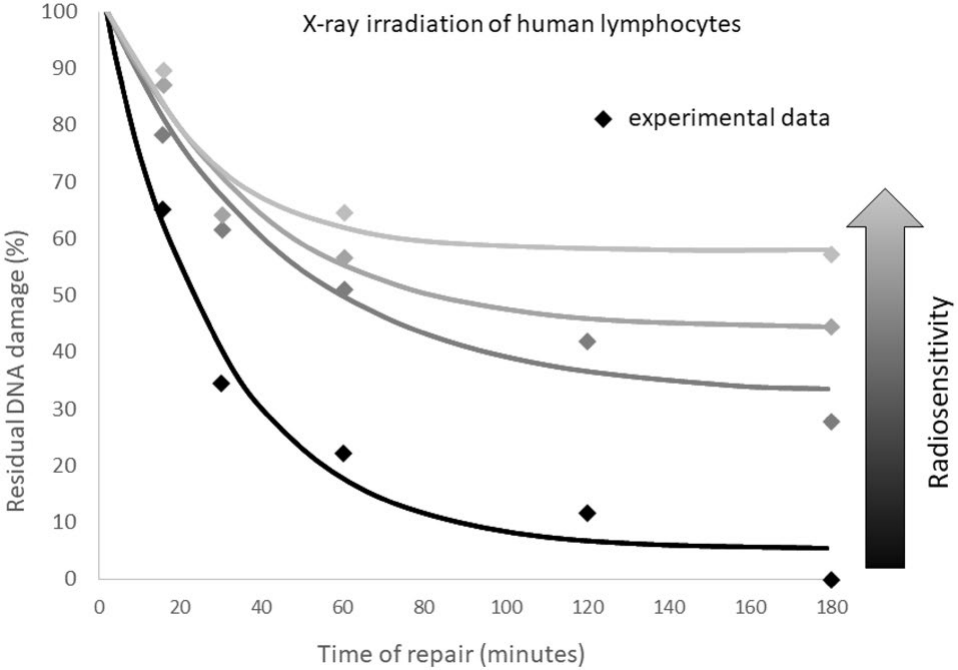
The residual DNA damage (lesions) represented by double-strand breaks (DSB) in human lymphocytes related to time, after X-ray irradiation of 2 Gy. Four different relationships represents four different sensitivities to radiation, from hyper-radiosensitivity (upper grey curve) to radioresistance (lower black curve)—this last case represents the strongest adaptive response effect (Fornalski 2019). The figure was created based on the paper by Müller et al. (2001) and presentation of Feinendegen (2012)

Let us assume that *N*_0,1_ denotes the initial number of damage (lesions) induced by dose *D*_1_ in moment zero (assumed that *N*_0,1_ = *μ*_0_ + *μ*_1_*D*_1_, see Eq. (5)). Moreover, dose *D*_2_ generates an additional number of damage, *N*_0,2_ = *μ*_1_*D*_2_, in moment Δ*t* (where *N*_0,2_ > *N*_0,1_).^1^ Figure 2 presents the time related probability of the adaptive response (*p*_AR_) and the number of unrepaired damage in time *N*(*T*) for a single dose *D*_2_ and the combination of priming and challenging doses, *D*_1_ + *D*_2_. Please note, that the *N*(*T*) relationship is a strongly decreasing function, however, it never goes to zero due to the hunchbacked shape of the adaptive response probability function: within our model, the stronger the repair processes are, the closer to zero the *N*(*T*) function is at a large *T*. This is consistent with experimental findings, see Fig. 3 based on the paper by Müller et al. (2001).

The Yonezawa (priming dose) experimental scheme, which is presented in Fig. 1, can be quantified as

7 $$ \delta = 1 - \frac{{N_{1 + 2} }}{{N_{2} }}, $$

where *N*_2_ corresponds to the general number of lesions (or mutations) in a single *D*_2_ scenario, while *N*_1+2_ is the general number of lesions (or mutations) in a *D*_1_ + *D*_2_ scenario. The delta parameter, *δ*, is the percentage difference between *N*_2_ and *N*_1+2_ (Fig. 1).

After some calculations, which are expressed in detail in the Appendix 1, Eq. (7) can be written as

8 $$ \delta = \frac{{N_{0,2} \left[ {1 - e^{{f\left( {D_{1} ,T} \right) - f\left( {D_{1} ,\Delta t} \right)}} } \right] - N_{0,1} e^{{f\left( {D_{1} ,T} \right) - f\left( {D_{1} ,0} \right)}} }}{{N_{0,2} }}, $$

where *f* are functions related to the adaptive response, see Appendix 1. Detailed forms of *f* functions are presented in Table 1.

**Table 1:** Detailed forms of functions *f* presented in Eqs. (8)–(11)

Function	No. of equation(s)	Solution
$f\left( {D_{1} ,T} \right)$	(8), (9)	$\frac{{\alpha_{0} }}{{\alpha_{2}^{3} }}D_{1}^{2} e^{{ - \alpha_{1} D_{1} - \alpha_{2} T}} \left( {\alpha_{2}^{2} T^{2} + 2\alpha_{2} T + 2} \right)$
$f\left( {D_{1} ,\Delta t} \right)$	(8), (9), (10)	$\frac{{\alpha_{0} }}{{\alpha_{2}^{3} }}D_{1}^{2} e^{{ - \alpha_{1} D_{1} - \alpha_{2} \Delta t}} \left( {\alpha_{2}^{2} \Delta t^{2} + 2\alpha_{2} \Delta t + 2} \right)$
$f\left( {D_{1} ,0} \right)$	(8), (9), (10), (11)	$2\frac{{\alpha_{0} }}{{\alpha_{2}^{3} }}D_{1}^{2} e^{{ - \alpha_{1} D_{1} }}$
$f\left( {D_{1} ,\Delta t_{1} + \Delta t_{2} } \right)$	(11)	$\frac{{\alpha_{0} }}{{\alpha_{2}^{3} }}D_{1}^{2} e^{{ - \alpha_{1} D_{1} - \alpha_{2} \left( {\Delta t_{1} + \Delta t_{2} } \right)}} \left[ {\alpha_{2}^{2} \left( {\Delta t_{1} + \Delta t_{2} } \right)^{2} + 2\alpha_{2} \left( {\Delta t_{1} + \Delta t_{2} } \right) + 2} \right]$
$f\left( {D_{2} ,\Delta t_{2} } \right)$	(11)	$\frac{{\alpha_{0} }}{{\alpha_{2}^{3} }}D_{2}^{2} e^{{ - \alpha_{1} D_{2} - \alpha_{2} \Delta t_{2} }} \left( {\alpha_{2}^{2} \Delta t_{2}^{2} + 2\alpha_{2} \Delta t_{2} + 2} \right)$
$f\left( {D_{1} ,\Delta t_{1} } \right)$	(11)	$\frac{{\alpha_{0} }}{{\alpha_{2}^{3} }}D_{1}^{2} e^{{ - \alpha_{1} D_{1} - \alpha_{2} \Delta t_{1} }} \left( {\alpha_{2}^{2} \Delta t_{1}^{2} + 2\alpha_{2} \Delta t_{1} + 2} \right)$
$f\left( {D_{2} ,0} \right)$	(11)	$2\frac{{\alpha_{0} }}{{\alpha_{2}^{3} }}D_{2}^{2} e^{{ - \alpha_{1} D_{2} }}$

See Appendix 1 for details of calculations

For simplicity, Eq. (5) can be approximated by the simple dependence of *N* *≈* *µ*_1_*D*, where background lesions, *µ*_0_, can be also neglected (*µ*_0_ << *µ*_1_). In that situation, Eq. (8) can be approximated by

9 $$ \delta = 1 - e^{{f\left( {D_{1} ,T} \right) - f\left( {D_{1} ,\Delta t} \right)}} - \frac{{D_{1} }}{{D_{2} }}e^{{f\left( {D_{1} ,T} \right) - f\left( {D_{1} ,0} \right)}} , $$

which is independent of parameters {*µ*} (Eq. 5).

The approach presented in Eqs. (8–9) describes the number of actual damage (lesions), still unrepaired, in the moment of *T*. This gives no information about mutations, which are created much later (>> *T*). One has to note, that Eq. (9) actually contains three free parameters (*α*_0_, *α*_1_, and *α*_2_) plus two time variables (Δ*t* and *T*, where 0 < Δ*t* < *T*), which are conditions of the dedicated experiment. In practice, however, the presence of the Yonezawa effect related to fast damage (lesions) is more difficult from a radiobiological point of view, because Eqs. (8–9) vary with time. Therefore, a more popular and representative situation is described by the second part (Chapter 4) of the proposed model, namely the Yonezawa effect on mutation frequency (late effects).

The parameter *δ* can vary from 1 (ideal protection and full reduction of all mutations) to some minimal value *δ*_min_ which can even be lower than zero (when the number of mutations after the *D*_1_ + *D*_2_ scenario is a little bit higher than the number of mutations after the single *D*_2_ scenario, but still lower than the sum of independent *D*_1_ and *D*_2_). The latter case can be therefore treated as a special case of the Yonezawa effect, however, in most studies it is assumed that *δ*_min_ = 0 (the case when the number of mutations after *D*_1_ + *D*_2_ scenario is the same as the number of mutations after single dose *D*_2_), see Appendix 1 for more information.

## Mutations in DNA

Here one considers the phenomena of mutations, which are permanent changes in DNA caused by mis-repaired lesions: all repair processes are finished now and all *N*(*T*) functions stabilized. That way one can consider Eq. (9), but for infinite time, *T* ⟶ ∞. This assumption means that the repair time is long enough to repair all possible lesions and only mutations are left. Thus, the analogous form of Eq. (9) becomes

10 $$ \delta = 1 - e^{{ - f\left( {D_{1} ,\Delta t} \right)}} - \frac{{D_{1} }}{{D_{2} }}e^{{ - f\left( {D_{1} ,0} \right)}} , $$

which describes the Yonezawa effect for mutations, namely late effects. Appendix 1 contains details of the above calculations. Both *f* functions are presented in Table 1.

Exemplary results of the parameter *δ* for the conditions from Fig. 2 are: *δ* = 0.725 for lesions (Eq. (9)) and *δ* = 0.728 for mutations (Eq. (10)), respectively. This latter case is presented in Fig. 1. One can generally see that the difference between lesions and mutations analyses is, within the scope of the model, generally marginal for a large *T* (> Δ*t*).

## Two priming doses

The single priming dose, *D*_1_, is just a special case. Another possible case can involve two similar priming doses: the first one *D*_1_, the second one (*D*_2_) received Δ*t*_1_ time after the first one, and the challenging dose (*D*_3_) received Δ*t*_2_ time after *D*_2_. One can note that *D*_1_ ≈ *D*_2_ < *D*_3_. It follows from Eq. (9) and Fig. 2b that the adaptive response of the second dose (similar to the first one) results in an apparent decrease of the *N*(*T*) function as compared to the one resulting from the *D*_2_ dose alone. Thus, one should expect that the challenging dose *D*_3_ given at a later time starts its repair functions from smaller number of lesions than when applied at Δ*t* after the first priming dose. In another words: two consecutive priming doses, given in proper time and value, can boost the repair process triggered by *D*_3_.

This approach needs more laborious calculations, which are presented in Appendix 1 as well. Finally, the parameter *δ* can be now defined as 1 − *N*_1+2+3_/*N*_3_, so

11 $$ \delta = 1 - e^{{ - f\left( {D_{1} ,\Delta t_{1} + \Delta t_{2} } \right) - f\left( {D_{2} ,\Delta t_{2} } \right)}} - \frac{{D_{2} }}{{D_{3} }}e^{{ - f\left( {D_{1} ,\Delta t_{1} } \right) - f\left( {D_{2} ,0} \right)}} - \frac{{D_{1} }}{{D_{3} }}e^{{ - f\left( {D_{1} ,0} \right) - f\left( {D_{2} ,0} \right)}} , $$

which describes the Yonezawa effect when the challenging dose *D*_3_ is applied after two priming doses *D*_1_ and *D*_2_. Additionally, *f* functions are presented in Table 1.

**Fig. 4:**
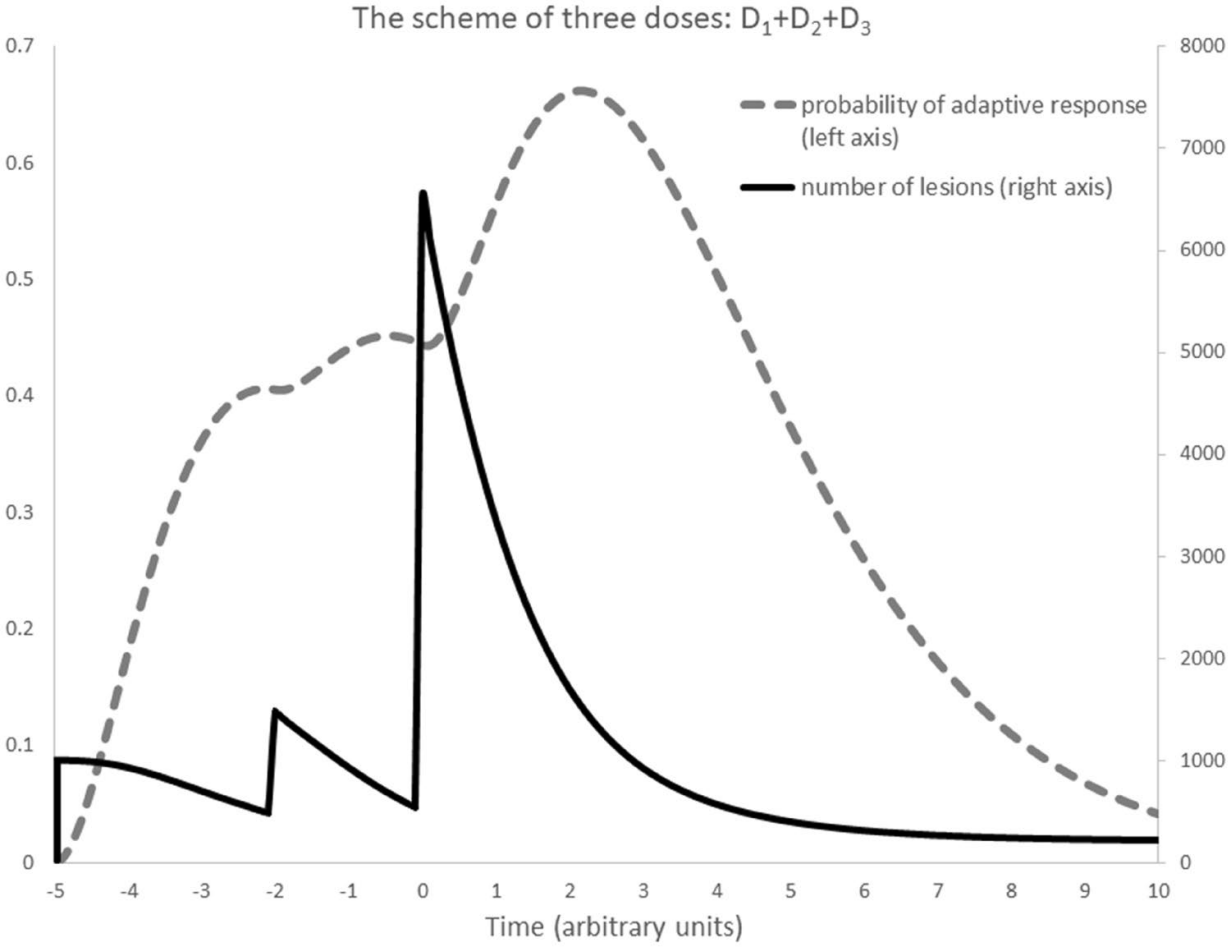
The case from the Fig. 2 with additional priming dose *D*_2_ = *D*_1_, received Δ*t*_1_ = 3 UT after *D*_1_. The challenging dose *D*_3_ was received Δ*t*_2_ = 2 UT after *D*_2_

Exemplary results of calculations, based on conditions from Fig. 2 with second priming dose, *D*_2_ = *D*_1_, are presented in Fig. 4.

Please also note, that Appendix 1 contains analogical calculations for multiple priming dose scenarios.

## Practical application and results

### Numerical methods

Both approaches, namely Eq. (9) for lesions and Eq. (10) for mutations, can be applied to experimental data to estimate the values of *α*_0_, *α*_1_ and *α*_2_ parameters. However, the differences in results obtained by those two equations are usually marginal for a large *T*, as discussed previously.

The application of the model, irrespective of the equation used, needs advanced numerical methods of non-linear optimization to estimate all input parameters based on experimental output. Here two of them were used: Simplified Genetic Algorithm (SGA) as well as Bounded Limited-memory Broyden–Fletcher–Goldfarb–Shanno (L-BFGS-B) one. Both methods and their applications are described in Appendix 2 in details.

### Lesions

**Fig. 5:**
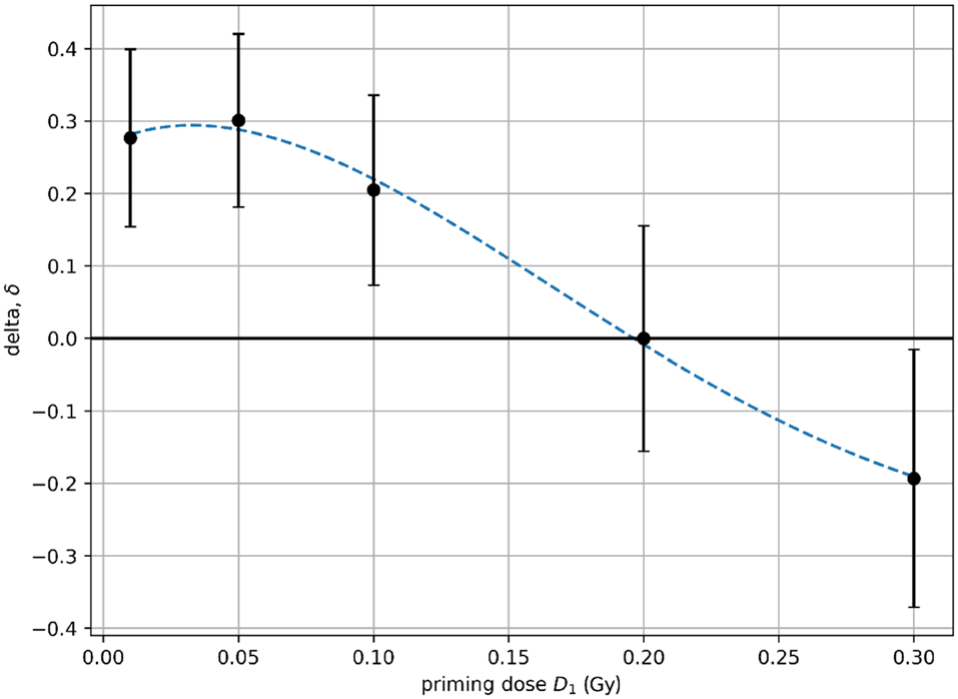
The relationship between the delta (*δ*) parameter for DNA lesions and the priming dose (*D*_1_) for human lymphocytes (Shadley and Wolff 1987), where *D*_2_ = 1.5 Gy, Δ*t* = 16 h, and *T* = 22 h. The Yonezawa effect disappears above approx. *D*_1_ = 200 mGy. The dashed line represents the potential purely empirical trend (best fit with *R*^2^ ≈ 0.997) given here by unsymmetrical Gaussian function: *f*(*x*) = exp(− (*x* − *a*)^2^/*b*) + *c* + (*x* − *a*)^(1/*d*)^, where *a* = − 0.036, *b* = 0.067, *c* = − 1.12, and *d* = 3.668

Equation (9) was applied to real experimental data where human lymphocytes were irradiated in vitro (X-ray) in three consecutive radiobiological researches conducted by Shadley and Collaborators (Shadley and Wolff 1987; Shadley et al. 1987; Shadley and Dai 1992). These studies were selected for analysis because of a large amount of data representing the Yonezawa effect. The first experiment (Shadley and Wolff 1987) studied the effect of 3-aminobenzamide (3AB) on the repair functions present during the adaptive response; additionally, the experiment studied the effect of the conditioning dose level on the priming dose effect. The lymphocytes for testing were taken from the peripheral blood of healthy 30–40 y.o. females. The blood was exposed to low doses at 32–34 h after stimulation, the challenging dose was given at 48 h. For one group, 3AB was applied immediately after the high dose. The results showed that application of 3AB immediately after the higher dose did reverse/eliminate the repairs of the adaptive response. Based on the results, low doses of 5, 10, 50, 100 mGy are capable of inducing an adaptive response when a higher dose is applied, while doses above 200 mGy do not have the ability to adapt human lymphocytes to ionizing radiation. This is perfectly illustrated in Fig. 5, where the priming dose *D*_1_ from the approximated range 10–100 mGy gives the highest value of *δ*.

The second analysed study (Shadley et al. 1987) considered the effect of the cell cycle phase during which certain doses are applied as well as how long the effect of a priming dose can last. Low doses were applied either before 2% phytohemagglutinin (PHA) addition (*G*_0_) or at times corresponding to *G*_1_, *S* or *G*_2_ phase of the first cell cycle. Higher doses are applied either in the same or the next cell cycle (40, 48, 66, 90, or 114 h after PHA addition).

**Fig. 6:**
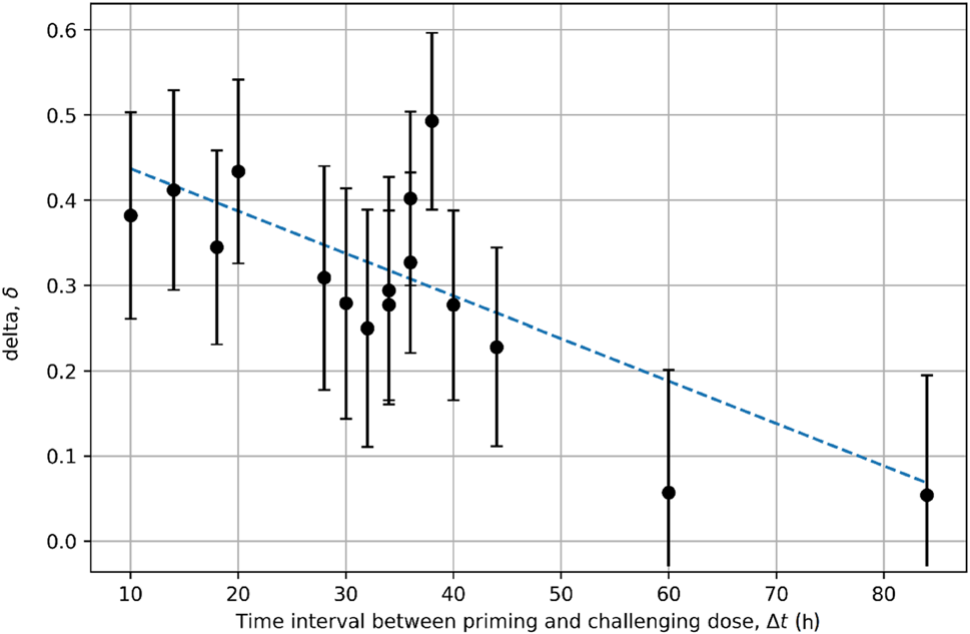
The relationship between the delta (*δ*) parameter for DNA lesions and the time interval between priming and challenging dose (Δ*t*) for human lymphocytes (Shadley et al. 1987), where *D*_1_ = 10 mGy, *D*_2_ = 1.5 Gy, and *T* − Δ*t* = 6 h. The dashed line represents the assumed potential linear trend (the best fit by *δ* = − 0.005 [h^−1^] Δ*t* + 0.487 with *R*^2 ^≈ 0.60) according to which the Yonezawa effect disappears above Δ*t* ≈ 100 h. The straight line was selected as the simplest best fit according to the existing scatter of data points. Due to potential outliers, this fitting was also tested by the robust Bayesian regression method (Fornalski et al. 2010) but the result was practically the same (*δ* = − 0.005 [h^−1^] Δ*t* + 0.483)

The collected data demonstrated how the response to low-dose radiation depends on whether the cells have been stimulated to divide. The results show that significantly fewer breaks were observed in cells pretreated with 0.01 Gy in *G*_1_, *S* or *G*_2_ than in those pretreated in *G*_0_. This suggests that the adaptation of cells to low doses requires mitogenic stimulation of lymphocytes. Concerning the lifespan of the adaptive response, data shows that lymphocytes treated with 1500 mGy at 40, 48, 66 h exhibited an adaptive response, and those treated later did not. Figure 6 shows the potential relationship between delta (*δ*) parameter and the time interval between priming and challenging dose (Δ*t*). One can deduce that for approx. Δ*t* > 100 h the Yonezawa effect completely disappears in human lymphocytes.

In the third analysed study (Shadley and Dai 1992), low doses were applied 12 h after culture (PHA application), at 18 h (first G_1_ phase after PHA) after stimulation the higher dose was applied. In this case, however, DNA mutations (namely, the chromosomal aberrations) were analysed but the experimental investigation was quite similar to the previous two studies (Shadley and Wolff 1987; Shadley et al. 1987) with a short *T*. Here, both aberration and deletion frequencies were reduced when a priming dose was applied, although there was a variability between the responses in samples from different donors. The results of this experiment show that G_1_ lymphocytes are capable of exhibiting a cytogenetic adaptive response.

Table 2 shows the exact raw data published in the three mentioned papers (Shadley and Wolff 1987; Shadley et al. 1987; Shadley and Dai 1992) to estimate the values of *α*_0_, *α*_1_ and *α*_2_ parameters using both numerical methods. The two first studies (Shadley and Wolff 1987; Shadley et al. 1987) can be simply treated as fully consistent and analysed jointly, because both of them show DNA lesions in the Yonezawa scheme. Thus, the results are:

- for the SGA: $\alpha_{0} = 22.9_{ - 4.0}^{ + 0.5}$ Gy^−2^ h^−3^, $\alpha_{1} = 79.4_{ - 11.2}^{ + 5.5}$ Gy^−1^ and $\alpha_{2} = 0.0832_{ - 0.0082}^{ + 0.0093}$ h^−1^.
- for the L-BFGS-B algorithm: *α*_0_ = 22.9 Gy^−2^ h^−3^, *α*_1_ = 79.5 Gy^−1^ and *α*_2_ = 0.0832 h^−1^.

**Table 2:** The source data used for estimation of α_0_, α_1_ and α_2_ parameters, selected for DNA lesions and chromosomal aberrations, based on three studies by Shadley and Collaborators (Shadley and Wolff 1987; Shadley et al. 1987; Sahdley and Dai 1992) for human lymphocytes

Source of data/number of table in original paper		*D*_1_ (Gy)	*D*_2_ (Gy)	Δ*t* (h)	*T* (h)	*N*_2_	*N*_1+2_	δ^a^
Shadley and Wolff (1987)	Table II	0.01	1.5	16	22	83/200^b^	60/200^b^	0.277
		0.05	1.5	16	22	83/200^b^	58/200^b^	0.301
		0.1	1.5	16	22	83/200^b^	66/200^b^	0.205
		0.2	1.5	16	22	83/200^b^	83/200^b^	0.000
		0.3	1.5	16	22	83/200^b^	99/200^b^	− 0.193^d^
		0.4	1.5	16	22	83/200^b^	103/200^b^	− 0.241^d^
		0.5	1.5	16	22	83/200^b^	126/200^b^	− 0.518^d^
Shadley et al. (1987)	Table I	0.01	1.5	44	50	101/300^b^	78/300^b^	0.228
		0.01	1.5	40	46	101/300^b^	73/300^b^	0.277
		0.01	1.5	36	42	101/300^b^	68/300^b^	0.327
		0.01	1.5	34	40	101/300^b^	73/300^b^	0.277
	Table II	0.01	1.5	34	40	68/200^b^	48/200^b^	0.294
		0.01	1.5	32	38	68/200^b^	51/200^b^	0.250
		0.01	1.5	30	36	68/200^b^	49/200^b^	0.279
		0.01	1.5	28	34	68/200^b^	47/200^b^	0.309
		0.01	1.5	10	16	68/200^b^	42/200^b^	0.382
	Table III	0.01	1.5	14	20	68/200^b^	40/200^b^	0.412
		0.01	1.5	20	26	76/200^b^	43/200^b^	0.434
		0.01	1.5	38	44	71/200^b^	36/200^b^	0.493
	Table IV	0.01	1.5	18	24	84/200^b^	55/200^b^	0.345
		0.01	1.5	36	42	92/200^b^	55/200^b^	0.402
		0.01	1.5	60	66	88/200^b^	83/200^b^	0.057
		0.01	1.5	84	90	93/200^b^	88/200^b^	0.054
Shadley and Dai (1992)	Table I	0.05	2	6	30	95/100^c^	48/100^c^	0.495
		0.05	4	6	30	188/100^c^	143/100^c^	0.239
		0.05	2	6	30	90/100^c^	74/100^c^	0.178
		0.05	4	6	30	240/100^c^	166/100^c^	0.308
		0.05	2	6	30	69/100^c^	55/100^c^	0.203
		0.05	4	6	30	118/100^c^	65/100^c^	0.449
		0.05	2	6	30	106/100^c^	87/100^c^	0.179
		0.05	4	6	30	218/100^c^	176/100^c^	0.193
		0.05	2	6	30	58/100^c^	51/100^c^	0.121
		0.05	4	6	30	192/100^c^	147/100^c^	0.234

^a^Calculated according to Eq. (7)

^b^Number of chromatid and isochromatid breaks/number of cells examined

^c^Number of chromosome aberrations (dicentrics, rings and deletions)/number of cells examined

^d^Negative values do not represent the Yonezawa effect, but they are useful for model calibration

The results obtained by both algorithms are qualitatively the same within the range of uncertainties, therefore both numerical methods work well and they can be treated on equal footing. In the case of the SGA, the uncertainties were estimated by the upper-lower bound method. Simulations were run for each worst case scenario assuming that numbers of lesions or mutations measured in experiments follow a Poisson distribution. As explained in the Appendix 2, in the L-BFGS-B algorithm it was not possible to calculate uncertainties of the parameters.

In the next step, one can observe that the data and results of all three cited papers (Shadley and Wolff 1987; Shadley et al. 1987; Shadley and Dai 1992) are fully comparable, regardless of chromosomal aberrations analysis in the last one. Therefore, they are able to be treated as a meta-data for joint analysis, which is carried out in the “Discussion”.

### Mutations

The analogical numerical research for mutations only can be investigated using Eq. (10). For this case, the comprehensive experimental data collected by Day et al. (2006, 2007) were used (see Table 3) because they offer a large combination of situations to be used in numerical analysis. In the study by Day et al. (2007), *Atm* knockout heterozygous pKZ1 mice were treated by a whole-body X-irradiation with a priming dose (*D*_1_) and, after a 4 h interval, a challenging dose (*D*_2_). 3 days after exposure, the mice were sacrificed, their spleen and prostate removed and snap-frozen until needed for scoring of chromosome inversions. Both the 0.01 and 10 mGy priming doses caused a similar in magnitude adaptive response to the challenging dose of 1 Gy, although a single dose of 0.01 mGy seemed to induce a chromosomal inversion frequency by itself. Based on the results by Day et al. (2007), being a *Atm* knockout heterozygote (Hishiya et al. 2005) does not affect the response to single low radiation doses (used here) or the induction of an adaptive response for inversions.

**Table 3:** The source data used for estimation of *α*_0_, *α*_1_ and *α*_2_ parameters, selected for DNA mutations, based on studies by Day et al. (2006, 2007) for chromosomal inversions in mice’s spleen and prostate

Source	Tissue	*D*_1_ (mGy)	*D*_2_ (mGy)	Δ*t* (h)	*T* (h)	*N*_2_ frequency (·10^–3^)	*N*_1+2_ frequency (·10^–3^)	δ^a^
Day et al. (2006)	Prostate	0.001	1000	4	72	5.72	0.93	0.837
		0.01	1000	4	72	5.72	1.70	0.703
		1	1000	4	72	5.72	1.88	0.671
		10	1000	4	72	5.72	0.98	0.829
Day et al. (2007)	Prostate	0.01	1000	4	72	4.66	0.88	0.811
		10	1000	4	72	4.66	1.13	0.758
	Spleen	0.01	1000	4	72	3.15	0.77	0.756
		10	1000	4	72	3.15	0.98	0.689

^a^Calculated according to Eq. (7)

The second analysed study (Day et al. 2006), used the very low priming doses of 0.001, 0.01, 1 or 10 mGy, which were administered 4 h before a challenge dose of 1 Gy. Mice were sacrificed 3 days after treatment to detect pKZ1 chromosomal inversion assay in the prostate. The results show that the 1–10 mGy priming doses reduced chromosomal aberration (inversion) frequency to below the spontaneous frequency level. The 0.001 mGy single priming dose had no significant effect, while the 0.01 mGy single priming dose increased the inversion frequency. Despite this, all four low and ultra-low priming doses caused an adaptive response when a 1 Gy challenging dose was administered (Day et al. 2006), which is presented in Table 3.

Both sets of data, for spleen and prostate postradiation mutation frequency in mice, show a strong Yonezawa effect (*δ* > 0.6). The calculated parameters are: *α*_0_ = 11160 Gy^−2^ h^−3^, *α*_1_ = 1400.9 Gy^−1^ and *α*_2_ = 0.0116 h^−1^ for spleen mutations (Day et al. 2007), and *α*_0_ = 15.92 Gy^−2^ h^−3^, *α*_1_ = 1148.7 Gy^−1^ and *α*_2_ = 0.00026 h^−1^ for prostate mutations calculated jointly for both studies (Day et al. 2006, 2007) (Table 3).

As mentioned earlier, the human lymphocytes chromosomal aberration data from Table 2 (Shadley and Dai 1992) are also related to mutations analysis. These data results in: *α*_0_ = 89.23 Gy^−2^ h^−3^, *α*_1_ = 174.86 Gy^−1^ and *α*_2_ = 1.41·10^–6^ h^−1^, which differ from the results for lesions.

## Discussion

The role of the time interval between two consecutive doses of ionizing radiation has been pretty well known for years and is the basis of e.g. dose fractionation. But the first experimental finding that this time interval is related to repair processes was conducted in 1940s by Prof. John R. Raper during his works in Manhattan Project (Raper 1947). At that time he irradiated groups of mice using beta radiation and observed a “recovery from the radiation damage” when the *conditioning* sublethal dose was applied prior to the *test* lethal dose. Raper’s experiments were repeated a few years later and showed the same effect but no detailed explanation was proposed (Cronkite et al. 1950). Next, in the late 1950s, the historical Elkind-Sutton experiments were conducted (Elkind and Sutton 1959). Elkind and Sutton irradiated mammalian cells with two doses, which they called *conditioning* and *test* doses, analogically to Raper’s terminology. They found that the time delay between doses can change the shape of the survival curve of irradiated cells but both doses were large and no low conditioning dose was tested.

Important changes appeared in 1980s and 1990s, when many studies began reporting that the radiation adaptive response effect showed strong results with a low priming dose and high challenging dose scheme (Olivieri et al. 1984; Shadley and Wolff 1987; Shadley et al. 1987; Shadley and Dai 1992; Yonezawa et al. 1990; Fan et al. 1990; Liu et al. 1992; Farooqi and Kesavan 1993; Cai et al. 1994). In the 1990s, this special case of adaptive response was called the priming dose effect or, a few years later, the Yonezawa effect (Wang et al. 2013, 2018, 2021; Liu et al. 2019). Today, we can also call it the Raper-Yonezawa effect to account for some historical aspects surrounding it. Anyway, from the experimental point of view, the easiest way to test the adaptive response appearance is the priming dose scheme: the first small dose, called the priming or conditioning dose, and some time later the higher one, called challenging dose. That situation results in smaller detrimental effects than when the same big dose is given as a single dose – of course only in situations when the radiation adaptive response was activated.

In the XXI century there have been several biomathematical models which have tried to explain, among others, the priming dose effect (Schöllnberger et al. 2001; Smirnova and Yonezawa 2003, 2004; Esposito et al. 2010; Zhao and Ricci 2010; Wodarz et al. 2014; Devic et al. 2018, 2020; Bondarenko et al. 2021), however, they are usually based on a set of differential equations, and in most cases they are not deeply related to the adaptive response phenomenon. The presented paper intends to explain the biophysical origin of the Yonezawa effect within the scope of a relatively simple model used so far in many of our papers (Fornalski et al. 2017; Dobrzyński et al. 2016, 2019). The considerations concern the effect of the use of a high challenging dose after a rather low priming dose given earlier. In the original papers by Yonezawa and coworkers (Yonezawa et al. 1990, 1996; Matsubara and Yonezawa 2004; Matsumoto et al. 2004; Smirnova and Yonezawa 2003, 2004), originally for mice, the challenging dose was so high that it caused the systematic death of mice with time. However, when the priming dose was added, this process was substantially hindered.

The proposed new model describing the Yonezawa effect contains only three free parameters. This model, however, is based on the radiation adaptive response probability function which makes it more grounded in biophysics. The model was validated on a group of experimental data from papers by Shadley et al. (Shadley and Wolff 1987; Shadley et al. 1987; Shadley and Dai 1992) and Day et al. (2006; 2007). Those studies represent the most broad and comprehensive data in the Yonezawa scheme, which is necessary for proper parameter calculations to validate the model. Other exemplary papers, which presented the effect (Fan et al. 1990; Liu et al. 1992; Farooqi and Kesavan 1993; Cai et al. 1994), contain too few results to be valuable for the model’s validation.

In practice, the model is reduced to a relatively simple equation, which connects the delta (*δ*) parameter (Fig. 1) with priming and challenging doses, and the radiation adaptive response probability function. This equation, however, is presented in two versions: for DNA lesions, namely fast damage (Eq. 9), and mutations, which are rather later effects (Eq. 10). The quantitative results obtained by Eqs. (9) and (10) are very similar, therefore one can use one equation (Eq. 9) for both types of data to improve and unify the model’s parameters, as well as to reduce some of uncertainties. We are aware that the number of lesions a long time after application of the challenging dose may only approximate the number of mutations. Therefore our model apparently simplifies the real situation in which the kinetics of late repairs should be better described.

We have to note that it is known (Berthel et al. 2019; Feinendegen et al. 2007; Mezentsev and Amudson 2011; Long et al. 2007; Ding et al. 2005) that low and high doses trigger separate groups of genes. As stated by Mezentsev and Amudson (2011), “Accumulating data suggest that the biological responses to high and low doses of radiation are qualitatively different”, that may be linked to the activation of “radiation-responsive genes after high- and low-dose exposures”. Therefore, our model in which the adaptive response at both doses is described by the same function of dose and time may not be adequate for a full explanation of the originally observed Yonezawa effect. This puts a natural limit on the value of the challenging dose. On the other hand, the alpha parameters including their uncertainties, which are connected with the biology of the studied object, endpoints, and proper timing of the whole process as well, may be so individual-dependent that the whimsical behaviour of Yonezawa effect can be expected.

The dose relationship presented by Eq. (9) has to be commented on here as well. Certainly, the right-hand side of Eq. (9) is not appropriate for very high doses *D*_2_ because it tends to zero for that case. This problem is much wider: firstly, a very high value of *D*_2_ would be lethal, thus no Yonezawa effect may be observed because of the organism's death. Secondly, as mentioned above, a very high value of *D*_2_ activates different groups of genes responsible for DNA repair, which makes the radioadaptation more complicated and this is not reflected in our relatively simple model. Therefore, the presented approach has a very important limitation: the model works well when the challenging dose, *D*_2_, is not loo large.

The alpha parameters calculated for all the data in Table 2 are as follows:

$$ \alpha_{0} = 36.2_{ - 8.0}^{ + 8.3} \; {\text{Gy}}^{{ - {2}}} {\text{h}}^{{ - {3}}} ,\;\alpha_{1} = 120.2_{ - 1.5}^{ + 2.6} \;{\text{Gy}}^{{ - {1}}} {\text{and}}\;\alpha_{2} = 0.0845_{ - 0.0060}^{ + 0.0085} \;{\text{h}}^{{ - {1}}} . $$

These results are related to human lymphocytes (Shadley and Wolff 1987; Shadley et al. 1987; Shadley and Dai 1992) and their potential radioadaptation, which reaches its maximum for the priming dose of *D*_1_ = 2*/α*_1_ ≈ 25.2 mGy and Δ*t* = 2*/α*_2_ ≈ 24 h after its exposure. It is worth mentioning here that in the original Yonezawa and Smirnova mice model (Smirnova and Yonezawa 2003, 2004), their theoretical consideration of the biological mechanisms responsible for the modifications of radiosensitivity is due to the change of radiosensitivity of the hematopoietic system. Consistently, the blood-forming system's cells were the objects of the studies (Smirnova and Yonezawa 2004).

**Fig. 7:**
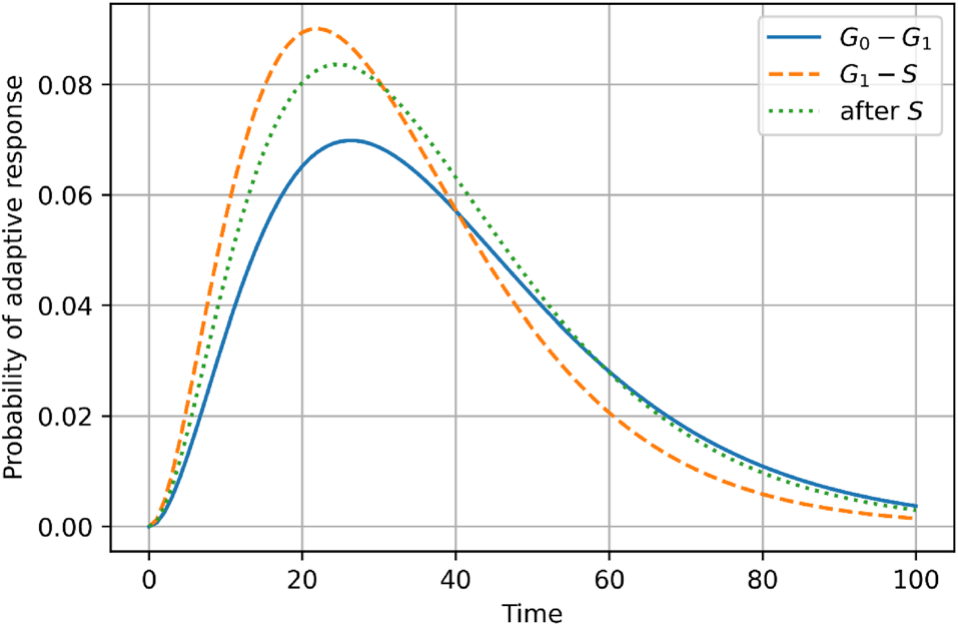
The non-normalized probability functions of radiation adaptive response in human lymphocytes (Shadley et al. 1987) in phase G_0_–G_1_ (blue solid line), in phase G_1_–S (orange dashed line) and after phase S (green dotted line), related to time (hours). The orange dashed line corresponds to the lowest radiosensitivity of the cell and therefore the strongest radioadaptation (color figure online)

The next finding, which can be observed in the detailed data from Shadley et al. studies (Shadley et al. 1987), is that the level of radioadaptation is connected with the cell cycle phase and its radiosensitivity. The analysis of the data shows that the strongest radiation adaptive response generated by the priming dose is connected with the lowest radiosensitivity of the cell, which seems to be quite natural (Fig. 7). It has long been known that cells in different cell cycle stages display different sensitivity to radiation. Cells in the late S phase are usually most radioresistant and cells in the M phase are most radiosensitive. A cellular response to DNA-damaging agents correlates not only with DNA replication and chromosome segregation stage. It also depends on the activation of the repair pathway maintaining the genetic integrity and activation of the cell cycle checkpoint. This complex mechanism is driven by the variety of key proteins concentrated in the cell (such as tumour suppressor proteins p53, inhibitor proteins p21, CHK kinases, ataxia telangiectasia-mutated ATM or ataxia-telangiectasia and RAD3-related ATR) and their inner cell signalling (Pawlik and Keyomarsi 2004).

After a cell enters cycle arrest two major DNA double-strand break repair pathways can occur: error prone NHEJ (Non-Homologous End Joining, dominant in G_1_/S phase) and HR (Homologous Recombination, present in late S or G_2_ phase). The greater proportion of error-free repair in late S phase may explain its radioresistance (lower radiosensitivity). If the DNA damage is fully repaired, the cell cycle continues. The choice of which pathway becomes activated is determined by the conflict between maintenance and resection of the DNA ends (Maier et al. 2016).

The differences in radioadaptation are, however, relatively small but nonetheless, the adaptive response in phase G_0_–G_1_ and after S (G_2_) is smaller than in G_1_-S, which is clearly presented in Fig. 7. The mechanisms explaining such cell cycle effects on the adaptive response to ionizing radiation are not fully understood, but it has been suggested that the adaptive response phenomenon may be due to cell cycle changes (Cramers et al. 2005) or arrest (Syljuåsen 2019) caused by the low priming dose of radiation. Since sensitivity to radiation varies with cell cycle stage, changes in cell cycle distribution may be responsible for the radiation adaptive responses (Hafer et al. 2007). Recently, many studies have been focussing on pointing out which of the repair pathway components are taking majority in radioadaptation induction (Hafer et al. 2007; Hendrikse et al. 2000; Boothman et al. 1998).

**Fig. 8:**
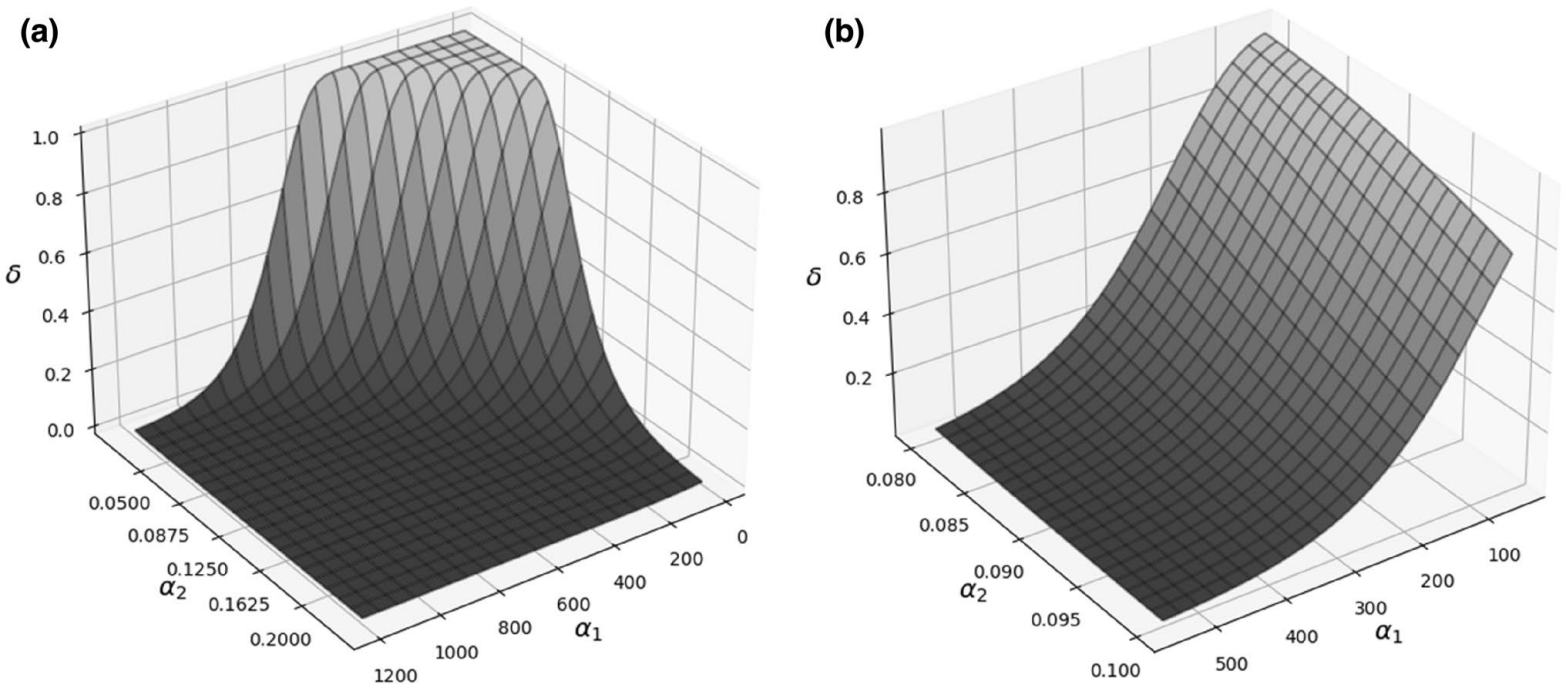
**a** The shape of delta (*δ*) parameter function from Eq. (9) for the exemplary sets of parameters and their ranges: *α*_0_ = 36.21 Gy^−2^ h^−3^, *α*_1_ from 20 to 1200 Gy^−1^, *α*_2_ from 0.02 to 0.23 h^−1^, *D*_1_ = 10 mGy, *D*_2_ = 1.5 Gy, Δ*t* = 34 h; plot **b** represents the same situation but two parameters’ ranges were narrowed: *α*_1_ from 50 to 550 Gy^−1^, and *α*_2_ from 0.08 to 0.1 h^−1^

The delta (*δ*) parameter, when the input data are too weak, can hardly be determined because less than 3 equations for 3 variables {*α*_0_, *α*_1_, *α*_2_} would give an infinite number of solutions. This is the reason why advanced numerical methods were necessary to calculate the model’s parameters. The delta (*δ*) parameter, however, is very sensitive to even the smallest changes of alphas, which display the Yonezawa effect in some ranges only. This is clearly illustrated in Fig. 8, where the presented surface can easily reach maximal or minimal values of *δ*.

Indeed, the uncertainties of the alpha parameters inferred from the data shown in Table 2 indicate the high selectivity of the model used in the description of the Yonezawa effect. However, the calculated alpha parameters can be useful for the prediction of delta factor for other dose schemes, which can be planned in future experiments. Moreover, this method can be useful for the radiation adaptive response probability function assessment (like Fig. 7) for e.g. cell behaviour modelling during irradiation.

One has to note that the presented results were calculated for experimental cases where the Yonezawa effect was pretty well observed. But this is not always the case: this effect is observed in approx. 50% of all expected cases (Tapio and Jacob 2007), which means that the radiation adaptive response appearance is quite a selective process: the probability that this phenomenon appears equals approx. 0.5, but when it does appear, it can be described by the *p*_AR_ distribution with proper {*α*} parameters.

The last item to discuss is terminology: it is often found in scientific literature that the priming dose effect (which can be called the Yonezawa—or Raper-Yonezawa—effect) is practically equivalent to the radiation adaptive response phenomenon. However, this is not the case: the priming dose (Yonezawa) effect is just a special case of the adaptive response with a specific dose fractionation scheme. Another examples of the adaptive response is constant low-dose rate irradiation (Dobrzyński et al. 2016) or so called “radiation training” by many small dose pulses (Socol et al. 2020), see Appendix 1 (“Mutations in DNA”). Nevertheless, year by year we learn more about radioadaptation (UNSCEAR 2000; Tapio and Jacob 2007; Guéguen et al. 2019) therefore it is high time for its wide biophysical and mathematical description, at least of the most popular priming dose scenario.

## Conclusions

The presented paper uses the radiation adaptive response theory to create a single equation (Eqs. (9) or (10)) for the Raper-Yonezawa (priming dose) effect (Fig. 1) when *D*_2_ is not too high, and the adaptive response probability function after *D*_2_ can be described by same formula as the one after the priming dose *D*_1_. The delivered equations were confronted with a group of experimental data on humans and mice to calculate the exemplary input parameters. For example $\alpha_{0} = 22.9_{ - 4.0}^{ + 0.5}$ Gy^−2^ h^−3^, $\alpha_{1} = 79.4_{ - 11.2}^{ + 5.5}$ Gy^−1^ and $\alpha_{2} = 0.0832_{ - 0.0082}^{ + 0.0093}$ h^−1^ are related to the human lymphocytes DNA lesions reduction in Yonezawa scheme. The relatively narrow range of these parameters indicates that their values may be strongly dependent on the individual case. Indeed, it is a set of parameters for one type of cells and their individual radiosensitivity. Therefore, the presented analysis shows that the level of radiation adaptive response is strictly connected with radiosensitivity (Fig. 7)—low radiosensitivity (so high radioresistance) is a result of a strong adaptive response of the cell, which is consistent with many recent findings presented in this paper. Finally, the proposed mechanistic model was quantified based on experimental data—e.g. lesions in human lymphocytes and chromosomal inversions in mice—to be able to predict the Raper–Yonezawa effect for future experimental and theoretical investigations.

## Appendix 1: Detailed calculations

## DNA lesions

Let us consider the relationship between repaired damage (lesions) and the adaptive response, d*N* = *−* *N* *p*_AR_ d*t*, see Eqs. (6) and (2). The simplest solution of that equation, as an indefinite integral, is represented by

12 $$ \int \frac{{{\text{d}}N}}{N} = - \int p_{{{\text{AR}}}} {\text{d}}t = - \int \alpha_{0} D^{2} t^{2} e^{{ - \alpha_{1} D - \alpha_{2} t}} {\text{d}}t, $$

so one can calculate it as

13 $$ - \int p_{{{\text{AR}}}} {\text{d}}t = \frac{{\alpha_{0} }}{{\alpha_{2}^{3} }}D^{2} e^{{ - \alpha_{1} D - \alpha_{2} t}} \left[ {\left( {\alpha_{2} t} \right)^{2} + 2\alpha_{2} t + 2} \right] \equiv f\left( {D,t} \right), $$

and assume such a solution as a function *f*(*D*,*t*). Thus, the definite integral has a form:

14 $$ - \mathop \int \limits_{a}^{b} p_{{{\text{AR}}}} {\text{d}}t = f\left( {D,b} \right) - f\left( {D,a} \right). $$

For example, after some calculations and integration for *N* from *N*_0_ to *N*(*T*) and for *t* from 0 to *T*, one can get

15 $$ N\left( T \right) = N_{0} e^{{ - \mathop \int \limits_{0}^{T} p_{{{\text{AR}}}} {\text{d}}t}} = N_{0} e^{{f\left( {D,T} \right) {-} f\left( {D,0} \right)}} . $$

This can be written in exact form as:

16 $$ N\left( T \right) = N_{0} \exp \left[ {\frac{{\alpha_{0} }}{{\alpha_{2}^{3} }}D^{2} e^{{ - \alpha_{1} D}} \left( {e^{{ - \alpha_{2} T}} \left[ {\left( {\alpha_{2} T} \right)^{2} + 2\alpha_{2} T + 2} \right] - 2} \right)} \right] $$

where *N*_0_ means the initial number of lesions (just after single *D* appearance, as described by Eq. (5)), *N*(*T*) is the actual remaining lesions, and *T* corresponds to the actual time. It should be noted, that Eq. (16) has an inverted Gompertzian-like shape in the function of time (Fornalski et al. 2020). Let us assume for further analyses, that the term

17 $$ \xi_{D} = 2\frac{{\alpha_{0} }}{{\alpha_{2}^{3} }}D^{2} e^{{ - \alpha_{1} D}} , $$

is constant for a dedicated dose pulse, *D*, which is also a constant value in exact conditions. One can also note, that *f*(*D*,0) = *ξ*_*D*_. Additionally, for further considerations, let us calculate the probability connected with the appearance of the adaptive response from dose *D* after some time Δ*t*:

18 $$ - \mathop \int \limits_{0}^{\Delta t} p_{{{\text{AR}}}} {\text{d}}t = f\left( {D,\Delta t} \right) - f\left( {D,0} \right) = \xi_{D} \left( {e^{{ - \alpha_{2} \Delta t}} \left[ {\frac{1}{2}\left( {\alpha_{2} \Delta t} \right)^{2} + \alpha_{2} \Delta t + 1} \right] - 1} \right), $$

which is an analogical solution to Eq. (16).

After the next period of time, from Δ*t* to *T*, the adaptive response from the same dose *D* is calculated as:

19 $$ - \mathop \int \limits_{\Delta t}^{T} p_{{{\text{AR}}}} {\text{d}}t = f\left( {D,T} \right) - f\left( {D,\Delta t} \right) = \xi_{D} \left\{ {e^{{ - \alpha_{2} T}} \left[ {\frac{1}{2}\left( {\alpha_{2} T} \right)^{2} + \alpha_{2} T + 1} \right] - e^{{ - \alpha_{2} \Delta t}} \left[ {\frac{1}{2}\left( {\alpha_{2} \Delta t} \right)^{2} + \alpha_{2} \Delta t + 1} \right]} \right\}. $$

However, when another dose is received in the moment Δ*t*, it is necessary to use Eq. (16) with time *T* shifted by Δ*t*:

20 $$ - \mathop \int \limits_{0}^{T - \Delta t} p_{{{\text{AR}}}} {\text{d}}t = f\left( {D,T - \Delta t} \right) - f\left( {D,0} \right) = \xi_{D} \left\{ {e^{{ - \alpha_{2} \left( {T - \Delta t} \right)}} \left[ {\frac{1}{2}\left( {\alpha_{2} \left( {T - \Delta t} \right)} \right)^{2} + \alpha_{2} \left( {T - \Delta t} \right) + 1} \right] - 1} \right\}. $$

In the scenario of the typical Yonezawa effect, one can consider the three moments in time mentioned above: (1) the first one, let us call it a moment zero, *t*_0_ = 0, when the first small priming dose (*D*_1_) was received, (2) the second one after a short period of time, Δ*t* > 0, when the challenging (much higher, *D*_2_ > *D*_1_) dose pulse was received, and (3) the third one, *T*, which corresponds to the actual moment, 0 < Δ*t* < *T*, when the number of lesions is measured.

Next, one can note that in the moment of Δ*t*, the number of damage (lesions) from dose *D*_1_ which remain unrepaired equals to (see Eq. (15)):

21 $$ N_{0,1}^{^{\prime}} = N_{0,1} e^{{f\left( {D_{1} ,\Delta t} \right) - f\left( {D_{1} ,0} \right)}} , $$

where *N*_0,1_ denotes the initial number of damage (lesions) induced by dose *D*_1_ in moment zero (assumed that *N*_0,1_ = *μ*_0_ + *μ*_1_*D*_1_, see Eq. (5)).

In time Δ*t*, dose *D*_2_ generates an additional number of damage, *N*_0,2_ = *μ*_1_*D*_2_, where *N*_0,2_ > *N*_0,1_ (because *D*_2_ > *D*_1_). Therefore, in the moment of Δ*t*, one needs to consider the total *N*_0,2_ + *N’*_0,1_ number of damage to repair. However, in the same moment, additional repair mechanisms from the second dose appear, which by assumption are additive to the first ones (because repair mechanisms from the first dose are still working). That way, in the later time, *T*, the number of unrepaired damage (lesions) equals:

22a $$ N\left( T \right) = \left( {N_{0,2} + N_{0,1}^{^{\prime}} } \right)e^{{f\left( {D_{1} ,T} \right) - f\left( {D_{1} ,\Delta t} \right) + f\left( {D_{2} ,T - \Delta t} \right) - f\left( {D_{2} ,0} \right)}} , $$

22b $$ N\left( T \right) = \left( {N_{0,2} + N_{0,1} e^{{f\left( {D_{1} ,\Delta t} \right) - f\left( {D_{1} ,0} \right)}} } \right)e^{{f\left( {D_{1} ,T} \right) - f\left( {D_{1} ,\Delta t} \right) + f\left( {D_{2} ,T - \Delta t} \right) - f\left( {D_{2} ,0} \right)}} , $$

which can be denoted as *N*_1+2_ for further considerations (see Eq. (7)). Let us note that as *D*_2_ increases, the value of $\xi_{D2}$ also increases with $D_{2}^{2}$, which causes a rapid decrease of *N*(*T*) (see analogical Fig. 3). To understand these relationships, Fig. 2 presents the time related probability of the adaptive response (*p*_AR_) and the number of unrepaired damage (lesions) in time *N*(*T*) for a single dose *D*_2_ and the combination of priming and challenging doses, *D*_1_ + *D*_2_.

Finally, at *N*_0,2_ > 0, the main quantification of the Yonezawa effect, the parameter *δ*, in the moment of *T* equals:

23 $$ \delta = 1 - \frac{{N_{1 + 2} }}{{N_{2} }} = 1 - \frac{{\left( {N_{0,2} + N_{0,1} e^{{f\left( {D_{1} ,\Delta t} \right) - f\left( {D_{1} ,0} \right)}} } \right)e^{{f\left( {D_{1} ,T} \right) - f\left( {D_{1} ,\Delta t} \right)}} }}{{N_{0,2} }}, $$

where *N*_2_ = *N*_0,2_ exp[*f*(*D*_2_,*T* *−* Δ*t*) *−* *f*(*D*_2_,0)]. One can note, that the denominator in the left-hand-side term of Eq. (23) represents the situation where the single dose *D*_2_ (without a priming dose) is given in the time *t*_0_ + Δ*t*, as shown in Fig. 2.

After some calculations Eq. (23) can be rewritten as:

24 $$ \delta = \frac{{N_{0,2} \left[ {1 - e^{{f\left( {D_{1} ,T} \right) - f\left( {D_{1} ,\Delta t} \right)}} } \right] - N_{0,1} e^{{f\left( {D_{1} ,T} \right) - \xi_{D1} }} }}{{N_{0,2} }}, $$

Assuming that *N* *≈* *µ*_1_*D*, because background lesions (*µ*_0_) can be neglected (*µ*_0_ << *µ*_1_), one can rewrite Eq. (24) as

25 $$ \delta = 1 - e^{{f\left( {D_{1} ,T} \right) - f\left( {D_{1} ,\Delta t} \right)}} - \frac{{D_{1} }}{{D_{2} }}e^{{f\left( {D_{1} ,T} \right) - \xi_{D1} }} , $$

which is independent of parameters {*µ*} (Eq. 5). It should be noted that Eqs. (24) and (25) are the same as Eqs. (8) and (9), respectively.

The parameter *δ* can vary from 1 to some minimal value *δ*_min_ which can even be lower than zero (but in most studies it is assumed that *δ*_min_ = 0). Generally, to keep the delta parameter above *δ* ≥ 0, one needs to fulfill the condition of

26 $$ f\left( {D_{1} ,\Delta t} \right) \ge - \ln \left( {e^{{ - f\left( {D_{1} ,T} \right)}} - \frac{{D_{1} }}{{D_{2} }}e^{{ - \xi_{D1} }} } \right), $$

which guarantees that the adaptive response signal from dose *D*_1_ will be still working when challenging *D*_2_ appears.

The Yonezawa effect completely disappears when the time distance (Δ*t*) between *D*_1_ and *D*_2_ is too large for the activation of repair mechanisms of the priming dose. Parameter *δ* attains its minimal (negative) value of

27 $$ \delta_{{{\text{min}}}} = \mathop {\lim }\limits_{\Delta t \to \infty } \delta = \frac{{ - D_{1} }}{{D_{2} }}e^{{ - \xi_{D1} }} < 0, $$

which is a clear information that in such a case the Yonezawa effect disappears.

## Mutations

Mutations are stable and unrepaired (or not properly repaired) lesions. Therefore, one can consider Eqs. (18), (19) and (20), but for infinite time, *T* ⟶ ∞. This assumption means that the repair time is long enough to repair all possible lesions and only mutations are left. Of course Eq. (18) will remain the same, but Eq. (19) will change into

28 $$ - \mathop \int \limits_{\Delta t}^{\infty } p_{{{\text{AR}}}} {\text{d}}t = f\left( {D,\infty } \right) - f\left( {D,\Delta t} \right) = - \xi_{D} e^{{ - \alpha_{2} \Delta t}} \left[ {\frac{1}{2}\left( {\alpha_{2} \Delta t} \right)^{2} + \alpha_{2} \Delta t + 1} \right] , $$

where *f*(*D*,*∞*) = 0. Analogically, Eq. (20) will change into

29 $$ - \mathop \int \limits_{0}^{\infty } p_{{{\text{AR}}}} {\text{d}}t = f\left( {D,\infty } \right) - f\left( {D,0} \right) = - \xi_{D} . $$

Thus, the analogous form of Eq. (25) becomes

30 $$ \delta = 1 - e^{{ - f\left( {D_{1} ,\Delta t} \right)}} - \frac{{D_{1} }}{{D_{2} }}e^{{ - \xi_{D1} }} , $$

which describes the Yonezawa effect for mutations, see equivalent Eq. (10).

The limitation for *δ*_min_, as the minimal possible value of the delta parameter, is the same as in the previous chapter, see Eq. (27), because both Δ*t* ⟶ ∞ and *T* ⟶ ∞. However, to keep *δ* ≥ 0 for mutations, one needs to fulfill the condition of

31 $$ f\left( {D_{1} ,\Delta t} \right) \ge - \ln \left( {1 - \frac{{D_{1} }}{{D_{2} }}e^{{ - \xi_{D1} }} } \right), $$

which is similar to Eq. (26).

## Two priming doses

The two priming doses case is presented in Fig. 4. After the first time interval (i.e. just before the moment of Δ*t*_1,_ so just before *D*_2_ appears) one can use reasoning analogous to the previous case: Eq. (18) can be rewritten as $- \mathop \int \nolimits_{0}^{{\Delta t_{1} }} p_{{{\text{AR}}}} {\text{d}}t$, which can be used analogically to the Eq. (22). After the second time interval (i.e. just before the moment of Δ*t*_1_ + Δ*t.*_2,_ so just before *D*_3_ appears), Eq. (19) can be substituted by $- \mathop \int \nolimits_{{\Delta t_{1} }}^{{\Delta t_{1} + \Delta t_{2} }} p_{{{\text{AR}}}} {\text{d}}t$, and Eq. (20) as $- \mathop \int \nolimits_{0}^{{\Delta t_{2} }} p_{{{\text{AR}}}} {\text{d}}t$; this can be used analogically to Eq. (22). However, after the appearance of *D*_3_ (we are considering mutations, therefore *T* = ∞), one shall calculate the adaptive response from the first dose ($- \mathop \int \nolimits_{{\Delta t_{1} + \Delta t_{2} }}^{\infty } p_{{{\text{AR}}}} {\text{d}}t$), second dose ($- \mathop \int \nolimits_{{\Delta t_{2} }}^{\infty } p_{{{\text{AR}}}} {\text{d}}t$) and the third one ($- \mathop \int \nolimits_{0}^{\infty } p_{{{\text{AR}}}} {\text{d}}t$, which equals $- \xi_{D}$, see Eq. (29)). The process of mutations repair can be written as:

32a $$ N_{1 + 2 + 3} = \left( {N_{0,3} + N_{1 + 2} } \right)e^{{f\left( {D_{1} ,\infty } \right) - f\left( {D_{1} ,\Delta t_{1} + \Delta t_{2} } \right)}} e^{{f\left( {D_{2} ,\infty } \right) - f\left( {D_{2} ,\Delta t_{2} } \right)}} e^{{f\left( {D_{3} ,\infty } \right) - f\left( {D_{3} ,0} \right)}} , $$

32b $$ N_{1 + 2 + 3} = \left[ {N_{0,3} + \left( {N_{0,2} + N_{0,1} e^{{f\left( {D_{1} ,\Delta t_{1} } \right) - \xi_{D1} }} } \right)e^{{f\left( {D_{1} ,\Delta t_{1} + \Delta t_{2} } \right) - f\left( {D_{1} ,\Delta t_{1} } \right) + f\left( {D_{2} ,\Delta t_{2} } \right) - \xi_{D2} }} } \right]e^{{ - f\left( {D_{1} ,\Delta t_{1} + \Delta t_{2} } \right) - f\left( {D_{2} ,\Delta t_{2} } \right) - \xi_{D3} }} . $$

The parameter *δ* can be now defined as 1 – *N*_1+2+3_/*N*_3_, so:

33 $$ \delta = 1 - e^{{ - f\left( {D_{1} ,\Delta t_{1} + \Delta t_{2} } \right) - f\left( {D_{2} ,\Delta t_{2} } \right)}} - \frac{{D_{2} }}{{D_{3} }}e^{{ - f\left( {D_{1} ,\Delta t_{1} } \right) - \xi_{D2} }} - \frac{{D_{1} }}{{D_{3} }}e^{{ - \xi_{D1} - \xi_{D2} }} , $$

which describes the Yonezawa effect when the challenging dose *D*_3_ is applied after two priming doses *D*_1_ and *D*_2_. Equation (33) is equivalent to the Eq. (11).

## Multiple priming doses

To present more general situation, let us consider *n* identical priming doses *D* separated by the same time shift Δ*t*. The situation where several single dose pulses are applied to an organism is called a “radiation training” (Socol et al. 2020). The challenging dose D* (where D* > *D*) is applied Δ*t* after the last of *n* doses *D* were applied.

Using the same reasoning as above, one can present the general equation for the parameter *δ* as:

34 $$ \delta = 1 - \frac{{\left[ {D^{*} + D\left( {1 + \left( {1 + \left( {1 + \cdots } \right)e^{{f\left( {D,\left( {n - 2} \right)\Delta t} \right) - \xi_{D} }} } \right)e^{{f\left( {D,\left( {n - 1} \right)\Delta t} \right) - \xi_{D} }} } \right)e^{{f\left( {D,{\text{n}}\Delta t} \right) - \xi_{D} }} } \right]e^{{ - f\left( {D,\Delta t} \right) - f\left( {D,2\Delta t} \right) - \cdots - f\left( {D,{\text{n}}\Delta t} \right)}} }}{{D^{*} }} $$

One can consider the situation where the number of dose pulses is infinite (*n* ⟶ ∞) and the time distance between them tends to zero (Δ*t* ⟶ 0). This situation is equivalent to chronic irradiation by a constant dose rate (*Ḋ* = const), which can be easily found in areas with high natural background radiation (Dobrzyński et al. 2015a, 2015b), for example. However, Eq. (34) would give an infinite level of adaptive protection ($\mathop {\lim }\limits_{\Delta t \to 0; n \to \infty } \delta = 1$), which is not possible. To avoid that problem, one should note that in this particular situation the probability function of the radiation adaptive response (Eq. (3)) saturates at some constant value:

35 $$ P_{c} = \mathop {\lim }\limits_{T \to \infty } p_{{{\text{AR}}}} \left( {\dot{D}} \right) = \frac{{2\alpha_{0} }}{{\alpha_{2}^{3} }}\dot{D}^{2} e^{{ - \alpha_{1} \dot{D}}} = \xi_{{\dot{D}}} , $$

which was originally calculated in (Dobrzyński et al. 2016), where *Ḋ* = const. This result will modify Eq. (16) to *N*(*T*) = *N*_0_ exp(*−* *P*_C_) = const which allows one to make analogical calculations as in previous subchapters. This is, however, not the subject of this paper, because chronic irradiation with constant dose-rate is not considered in the Yonezawa effect. Still, one can pose a question whether the natural radiation can function as a factor reducing possible effects of eventual higher doses (Mortazavi et al. 2012).

To conclude, the radiation adaptive response effect (namely, radioadaptation) is a wide phenomenon. Special case of radioadaptation is the priming dose effect (called the Raper-Yonezawa effect). Another example of the adaptive response is e.g. constant low-dose rate irradiation (see Eq. (35)), like in high background radiation areas (Dobrzyński et al. 2015a, 2015b).

## Appendix 2: Numerical methods

## Simplified Genetic Algorithm (SGA)

**Fig. 9:**
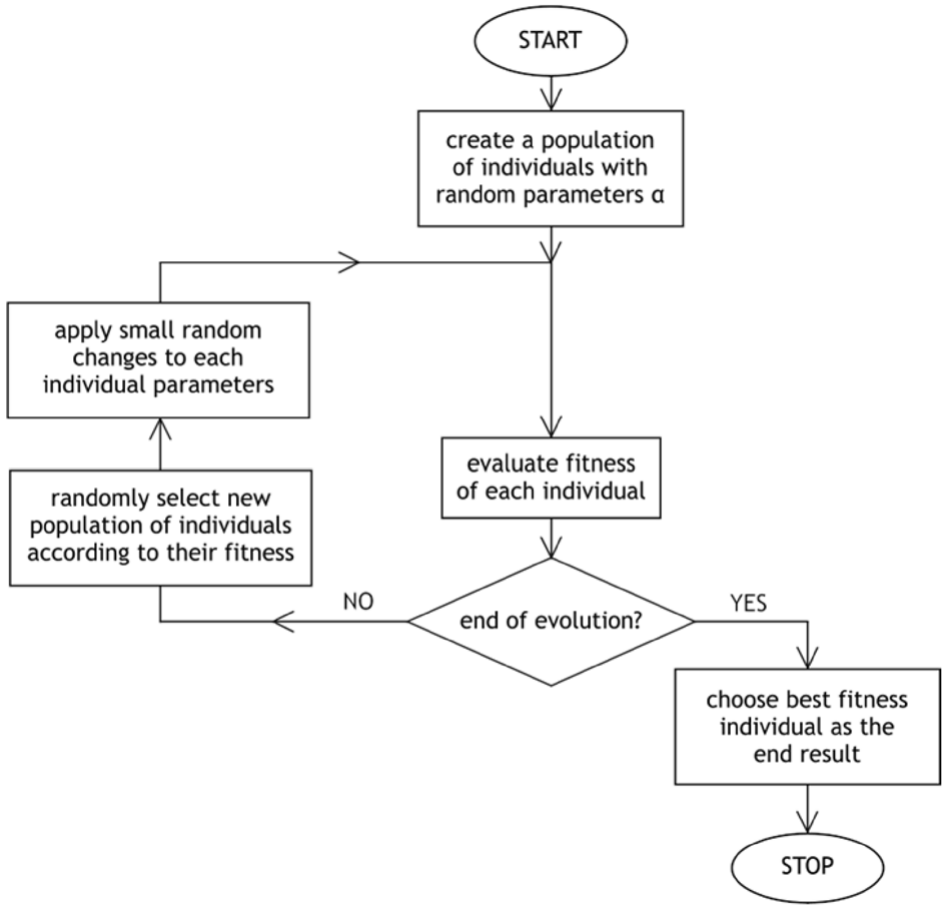
The flow chart of the Simplified Genetic Algorithm (SGA) used to evaluate parameters *α*_0_, *α*_1_ and *α*_2_

The proposed methodology starts with the well-known least squares method, where Eqs. (9) (or (10)) has defined the function to be fitted. This function is complicated, thus an iterative method of finding the minimum of sum of squared residuals had to be used. Thus, the most suitable method of parameters {*α*} assessment is the genetic algorithm. However, the use of the classical genetic algorithm (Banzhaf et al. 1998; Whitley 1994) is not suitable for finding a global minimum, for several reasons. One of them is limited possibility of coding a broad range of real numbers (such as parameters {α}) into artificial “genes” with keeping sufficient resolution. Another is that two steps of the classical genetic algorithm (namely crossover and mutation) have a tendency to change these parameters rapidly, resulting in an escape from the best solution neighbourhood. Significant changes to the algorithm were introduced including removal of the crossover step and applying small random changes directly to the parameters instead of randomly mutating artificial “genes”. A much simpler yet more effective algorithm mixing the features of genetic algorithms with particle swarm optimization^2^^,^ 3 and simulated annealing^4^ was then obtained (Fig. 9).

Simulation starts with creating a set (“a population”) of 99 individuals. Each individual represents all three-parameter values: *α*_0_, *α*_1_ and *α*_2_, chosen randomly as real positive numbers at the beginning of simulation. These values are used to calculate the fitness function of each individual, which in our case is reciprocal of the sum of squared residuals (each residual is the difference between *δ* measured in the experiment and *δ* calculated with given values of *α*_0_, *α*_1_ and *α*_2_). Next, a new set of individuals is selected from the old one with the probability of being chosen proportional to the fitness function of a given individual. This way individuals with the best fitness can be duplicated and these with the worst can be eliminated. Next, small random changes are applied to parameters in the new population, which becomes the current one. These steps make up one loop (“a generation”) of the algorithm, which is repeated until an end condition is fulfilled. The condition can be defined as being unable to find better parameters as the simulation progresses or simply reaching the maximum generation number, which in our case was 6,000,000.

Since there is a risk of not finding a global solution in a single simulation, simulations conducted by the described algorithm were run at least 50 times and manually checked for obvious mistakes (i.e. when the algorithm got stuck in a local solution or wasn't able to even get on the right path to global solution). These mistakes can be spotted by comparing the fitness functions of many simulations. In our case, it was safe to assume that if they differ more than a 1% from the best fitness function obtained in any simulation, the result should be ignored. Luckily, there were not many mistakes (less than 10%). Moreover, correct results were used to define the region of interest and more precise values of parameters were obtained by a brute-force search.^5^

## Bounded Limited-memory Broyden–Fletcher–Goldfarb–Shanno (L-BFGS-B) algorithm

The second approach also uses *χ*^2^ function to find the best estimation of alpha parameters. In this case, however, the optimization procedure uses a quasi-Newtonian algorithm called BFGS (Broyden–Fletcher–Goldfarb–Shanno) using a limited amount of computer memory with additional bound constraints of variables (Byrd et al. 1995; Zhu et al. 1997). L-BFGS-B method uses an estimate of the inverse Hessian matrix to drive its search in the variable space. The advantages of this algorithm are fast convergence and relatively low computational complexity. However, one should also be aware of its shortcomings: the L-BFGS-B algorithm may turn out to be divergent when the starting point is far from the solution sought and it does not guarantee finding the global minimum—it may happen that the parameters found only correspond to the local minimum. To minimize the risk that the found parameters would correspond to a local minimum instead of a global minimum, the algorithm was started from multiple points in the parameter space within a reasonable range. The L-BFGS-B algorithm should not be used for very flat functions. The limitations of numerical precision make it impossible to compute the gradient of such a function. This creates the last disadvantage: the calculated uncertainties of the fitted function’s parameters are inconsistent, thus the uncertainties were calculated by SGA method only.

## Footnotes

1 Please note, that *N*_0,2_ relationship intentionally omits *μ*_0_ parameter to avoid double counting of background lesions/mutations in Eq. (7).

2 http://www.scholarpedia.org/article/Particle_swarm_optimization.

3 https://www.sciencedirect.com/topics/engineering/particle-swarm-optimization.

4 https://www.sciencedirect.com/topics/engineering/simulated-annealing-algorithm.

5 https://www.oxfordreference.com/view/10.1093/oi/authority.20110803095532553.
